# The effect of hormone therapy on women's quality of life in the first year of the Estonian Postmenopausal Hormone Therapy trial

**DOI:** 10.1186/1756-0500-5-176

**Published:** 2012-04-03

**Authors:** Piret Veerus, Sirpa-Liisa Hovi, Tiina Sevón, Myra Hunter, Elina Hemminki

**Affiliations:** 1 Department of epidemiology and biostatistics, National Institute for Health Development, Hiiu 42, 11619, Tallinn, Estonia; 2 Hjelt Institute, University of Helsinki, PO BOX 40 (Kytösuontie 11), FI-00014 University of Helsinki, Helsinki, Finland; 3 National Institute for Health and Welfare (THL)/Finnish Office for Health Technology Assessment (Finohta), POB 30 (Lintulahdenkuja 4), FI-00271, Helsinki, Finland; 4 National Institute for Health and Welfare (THL), POB 30 (Lintulahdenkuja 4), FI-00271, Helsinki, Finland; 5 Dept Psychology, Institute of Psychiatry (Guy’s Campus), King’s College London, SE1 9RT, London, UK; 6 Tampere School of Public Health, FI-33014, University of Tampere, Tampere, Finland

**Keywords:** Randomised controlled trial, Postmenopausal hormone therapy, Quality of life

## Abstract

**Background:**

For postmenopausal women, the main reason to start hormone therapy (HT) is to reduce menopausal symptoms and to improve quality of life (QOL). The aim of this study was to analyse the impact of HT on different aspects of symptom experience and QOL during a randomised trial.

A total of 1823 postmenopausal women were recruited into the Estonian Postmenopausal Hormone Therapy (EPHT) trial in 1999–2001. Women were randomised to blind HT, open-label HT, placebo or non-treatment arm. After one year in the trial, a questionnaire was mailed and 1359 women (75%) responded, 686 in the HT arms and 673 in the non-HT arms. Mean age at filling in the questionnaire was 59.8 years. The questionnaire included Women's Health Questionnaire (WHQ) to assess menopause specific QOL of middle-aged women together with a 17-item questionnaire on symptoms related to menopause, a question about painful intercourse, and a question about women's self-rated health.

**Results:**

After one year in the trial, fewer women in the HT arms reported hot flashes, trouble sleeping, and sweating on the symptom questionnaire. According to WHQ, women in the HT arms had fewer vasomotor symptoms, sleep problems, and problems with sexual behaviour, but more menstrual symptoms; HT had no effect on depression, somatic symptoms, memory, attractiveness, or anxiety. A smaller proportion of women reported painful intercourse in the HT arms. There were no significant differences between the trial arms in women’s self-rated subjective health.

**Conclusions:**

The results from the EPHT trial confirm that HT is not justified for treating symptoms, other than vasomotor symptoms, among postmenopausal women. WHQ proved to be a useful and sensitive tool to assess QOL in this age group of women.

## Background

Based on results from long-term randomised trials, the main indication for hormone therapy (HT) among postmenopausal women is currently the treatment of menopausal symptoms [1]. At the same time, HT can have adverse outcomes, such as vaginal bleeding and breast tenderness [2], so its overall benefit on subjective well-being can be estimated by comparing symptom experience as well as the quality of life (QOL) of women using HT with those who do not.

The aim of the present study, which was part of the Estonian Postmenopausal Hormone Therapy (EPHT) trial, was to determine whether women receiving HT in a randomised trial experienced fewer symptoms and better QOL after one year of use than women receiving a matched placebo or no drug. Menopause specific QOL, assessed using the Women’s Health Questionnaire (WHQ) [3], was defined as women’s perceptions of their physical and emotional well being. Earlier, QOL has been reported in the EPHT trial at year two using EuroQoL [3] which is a generic health-related QOL measurement tool.

Women’s QOL at the end of the first trial year was assessed using a mailed questionnaire which included the Women’s Health Questionnaire (WHQ) [4], which has been developed to investigate psychological and somatic symptoms experienced by peri- and postmenopausal women and its psychometric properties have already been well documented [5,6]. In addition, information on self-rated health status, specific symptoms related to menopause and painful intercourse was compared among women in different trial arms.

In previous studies, the effect of HT on QOL among postmenopausal women has been studied in the WISDOM trial in the United Kingdom and in the PEPI trial and the WHI trial in the United States [2,7-9]. All these trials reported a positive effect of HT on vasomotor symptoms. The participants in the EPHT trial were younger than the women participating in the WISDOM and WHI trial, and the number of participants in the PEPI trial was relatively small. Despite the age differences the results from the EPHT Trial are consistent with previous findings from other trials.

## Methods

**Figure 1:**
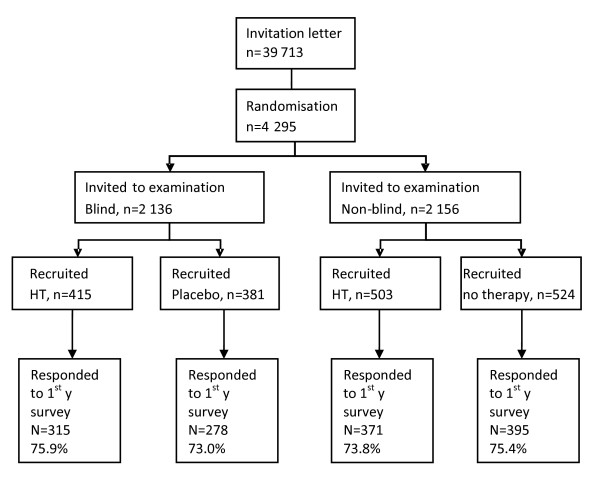
EPHT trial flow chart.

Women were recruited into the trial by means of a questionnaire sent to all 50-64 year old Estonian speaking women in two areas (Tallinn and Tartu and their surrounding counties) in 1999-2001 (n = 39,713) together with an invitation to participate in a randomised preventive HT trial. They were sent an information leaflet containing information on HT available at that time and a detailed description of the trial, inviting women to participate in this long-term trial studying the effects of HT on women’s health. The names and addresses were obtained from the Estonian population registry. A detailed description of the recruitment procedure, inclusion and exclusion criteria has been published elsewhere.[10] Women were asked about their education, living area, marital status, health status, last menstrual period (LMP), views about menopause, and 17 different symptoms that have been associated with menopause. Women who responded and were eligible according to the questionnaire data (n = 4,295) were randomised to one of the four trial arms: either to blind HT arm, placebo arm, non-blind HT arm or non-treatment arm (Figure 1), and then invited to the baseline assessment.

For the present analysis, women in the blind and the non-blind HT arms were combined and these arms were named HT arms, whereas women participating in the placebo and the non-treatment arm were analysed together and this group was named non-HT arms. From the 2,383 women who came to see the trial physician, 1,823 were recruited after secondary assessment of eligibility (918 to the HT arms and 905 to the non-HT arms).

Regardless their hysterectomy status (13% of women had undergone subtotal hysterectomy), women in the HT arms received 0.625 mg conjugated oestrogen (CEE) plus 2.5 mg medroxyprogesterone acetate (MPA), or 0.625 mg CEE and 5 mg of MPA if they were within 3 years from their last period. Women in the non-HT arms received either placebo or no treatment. At the end of the first trial year, nearly half of the women in the HT arms reported to have used more than 80% of their trial medication, and another 37% were of medium adherence using 10-79% of allocated treatment. In the non-HT arms, very few women (2% used it for more than 80% of trial duration) started HT, mainly due to menopausal symptoms [11].

After one year in the trial women received a postal questionnaire and 1,359 (74.5%) responded, 686 in HT and 673 in non-HT arms. Women were asked about symptoms related to menopause with a 17-item symptom list (the same as at baseline), and about menopause specific QOL using the WHQ, the same version as used in the UK in the WISDOM-trial [7]. Self-rated health status was to be ranked as very good, good, moderate, satisfactory, poor, or very poor.

The WHQ is a 36 item scale developed to measure emotional and physical symptoms in middle-aged women and it contains nine subscales. The WHQ was translated into Estonian by two Estonian researchers with good English skills. The feasibility of the translation was tested by a few Estonian lay women aged 50-65 years, and they had no difficulties completing on the questionnaire. Later, the questionnaire was back-translated into English by a native English speaking Estonian translator. The WHQ subscales include: depressed mood (7 items), anxiety/fears (4 items), sleep problems (3 items), somatic symptoms (7 items), menstrual symptoms (4 items), vasomotor symptoms (2 items), memory/concentration (3 items), attractiveness (2 items), sexual behaviour (3 items). The attractiveness subscale includes questions about self-esteem. The questionnaire answers have a 4-point scale: 1 = yes, definitely, 2 = yes, sometimes, 3 = no, not much and 4 = no, not at all. According to the scale author’s recommendation, item scores are transformed into a dichotomous scale: if the response is 1 or 2, it is scored 0. If the response is 3 or 4, it is scored 1. For each subscale the mean is calculated; 0 is interpreted as good and 1 as poor quality of life. For certain items the scoring is reversed as some items are worded positively and some negatively [6].

The statistical significance of the differences between the different arms was determined using *t*-test, *χ*^2^-tests, Wilcoxon rank sum tests, and odds ratios with 95% confidence intervals. All analyses were done by intention-to-treat principle. No adjustment was used during the statistical analysis. Correlation between sleeping problems and vasomotor symptoms was assessed by Pearson correlation coefficient. The software used was SAS version 9.

## Results

The overall response rate to the first year questionnaire was 74.5%. There were no differences between the trial arms in response rate. In the blind HT arm, 315 women (75.9%), in the placebo arm, 278 women (73.0%), in the non-blind HT arm, 371 women (73.8%), and in the non-treatment arm, 395 women (75.4%) returned the mailed questionnaire.

There were no differences between the respondents in different trial arms regarding their education (35% had a university degree), marital status (63% being married or cohabiting) or years since menopause (about one tenth of women were within 3 years from menopause) (Table 1). The mean age of respondents in the HT arms was statistically significantly lower than that in the non-HT arms (59.5 versus 60.1 years, SD 4.0), still the median age of respondents was the same in HT and non-HT arms and there was no difference between the trial arms as regards time since menopause. Also, the mean age of all women participating in the HT arms of the trial was a bit lower than those in the non-HT arms. Detailed baseline characteristics of trial participants are presented elsewhere [10].

**Table 1:** Comparison of first year survey respondents’ background characteristics in different trial arms, EPHT Trial

Background characteristic	Non-HT arms N=673	HT arms N=686	p-value
Age group, yrs			0.004^*^
50-54	86 (13%)	117 (17%)	
55-59	226 (34%)	261 (38%)	
60+	361 (54%)	308 (45%)	
Age, yrs			0.011^†^
mean	60.1	59.5	
median	60.5	60.5	
SD	4.0	4.0	
Education, yrs			0.213^*^
<= 12	450(67%)	436 (64%)	
>12	222 (33%)	248 (36%)	
Marital status			0.719^*^
married or co-habiting	420 (63%)	432 (63%)	
single	39 (6%)	46 (7%)	
divorced or widowed	208 (31%)	203 (30%)	
Duration of menopause, yrs			0.184^*^
<= 3	61 (9%)	77 (11%)	
>3	612 (91%)	608 (89%)	

^*^ χ^2^-test.

^†^ t-test.

The means for the WHQ scales were compared at the end of the first trial year in different arms (Table 2). Women in HT arms had better scores for vasomotor symptoms (p < 0.0001), sleep problems (p = 0.005) and sexual behaviour (p = 0.001). A separate analysis showed that women with vasomotor symptoms tended to report sleep problems (r = 0.28, p < 0.001), in HT arms (r =0.29, <0.001) as well as in non-HT arms (r = 0.25, p < 0.001).

**Table 2:** Quality of life according to mean scales of WHQ at the end of the first trial year, EPHT Trial

Variable	Non-HT arms	HT arms	p-value^*^	p-value^†^
	Mean (SE)	Mean (SE)		
Depressed mood	0.22 (0.01)	0.21 (0.01)	0.308	0.539
Somatic symptoms	0.40 (0.01)	0.39 (0.01)	0.212	0.282
Memory/concentration	0.42 (0.01)	0.41 (0.01)	0.447	0.490
Vasomotor symptoms	0.36 (0.01)	0.21 (0.01)	<0.0001	<0.0001
Anxiety/fear	0.27 (0.01)	0.27 (0.01)	0.519	0.642
Sexual behaviour	0.46 (0.02)	0.36 (0.02)	0.001	<0.001
Sleep problems	0.39 (0.01)	0.34 (0.01)	0.005	0.005
Menstrual symptoms	0.25 (0.01)	0.28 (0.01)	0.027	0.017
Attractiveness	0.53 (0.01)	0.53 (0.01)	0.811	0.791

^*^ Wilcoxon rank sum test.

^†^ t-test.

There was no difference in the mean WHQ scales for depressed mood, somatic symptoms, memory/concentration, anxiety/fears, and attractiveness subscales. However, women in HT arms had higher mean scales for menstrual symptoms (p = 0.02).

When asking about specific symptoms within past two weeks, fewer women in the HT arms reported sweating (OR = 0.69; 95%CI: 0.55-0.86), trouble sleeping (0.63;0.49-0.80), and hot flashes (0.28;0.21-0.37) at the end of the first trial year, compared with women in the non-HT arms (Table 3). There were no differences in the reporting of other symptoms. In general the symptoms that were most often reported by respondents were aches or stiffness in joints, lack of energy and backache.

**Table 3:** Number and proportion of women reporting symptoms within past two weeks at the end of the first trial year in different trial arms, EPHT Trial

Symptom	Non-HT arms	HT arms	p-value^*^	OR (95 % CI)^†^
Lack of energy	347 (51.6%)	379 (55.3%)	0.173	1.16(0.94-1.44)
Aches/stiffness in joints	374 (55.6%)	376 (54.8%)	0.778	0.97(0.78-1.20)
Backaches	264 (39.2%)	263 (38.3%)	0.737	0.96(0.77-1.20)
Headaches	235 (34.9%)	236 (34.4%)	0.842	0.98(0.78-1.22)
Irritability	210 (31.2%)	204 (29.7%)	0.557	0.93(0.74-1.18)
Sweating	254 (37.7%)	202 (29.5%)	0.001	0.69(0.55-0.86)
Trouble sleeping	220 (32.7%)	160 (23.3%)	<0.001	0.63(0.49-0.80)
Diarrhoea or constipation	155 (23.0%)	158 (23.0%)	1.000	1.00(0.78-1.29)
Feeling blue or depressed	142 (21.1%)	149 (21.7%)	0.780	1.04(0.80-1.34)
Dizzy spells	149 (22.1%)	139 (20.3%)	0.397	0.89(0.69-1.16)
Water retention	97 (14.4%)	110 (16.0%)	0.405	1.13(0.84-1.53)
Sore throat	67 (10.0%)	88 (12.8%)	0.096	1.33(0.95-1.86)
Hot flashes	219 (32.5%)	82 (12.0%)	<0.001	0.28(0.21-0.37)
Shortness of breath	90 (13.4%)	85 (12.4%)	0.589	0.92(0.67-1.26)
Persistent cough	68 (10.1%)	69 (10.1%)	0.978	1.00(0.70-1.42)
Upset stomach	85 (12.6%)	72 (10.5%)	0.218	0.81(0.58-1.13)
Loss of appetite	22 (3.3%)	20 (2.9%)	0.707	0.89(0.48-1.65)

^*^ χ^2^-test.

^†^ Non-HT arms as a reference group.

At the end of the first trial year, very few women complained about painful intercourse; those in HT arms reported painful intercourse less frequently than women in the non-HT arms (p < 0.04), while the number of women who were not sexually active (about one quarter) was nearly the same in all arms of the trial (Table 4).

**Table 4:** Number and proportion of women reporting painful intercourse during the first trial year in different trial arms, EPHT Trial

	Non-HT arms	HT arms	p-value^*^
Pain	57 (8.6%)	35 (5.2%)	0.045
No pain	427 (64.5%)	445 (66.0%)	
No intercourse	178 (26.9%)	194 (28.8%)	

^*^ χ^2^-test.

There were no differences between the trial arms in self-rated health status (Table 5). More than one third of women stated their health was good or very good, and more than half moderate or satisfactory. The number of women who stated their health to be poor or very poor was very small.

**Table 5:** Women’s self-rated health status in different trial arms at the end of the first trial year, EPHT Trial

	Non-HT arms	HT arms	p-value^*^
			0.423
Very good, good	219 (33.3%)	239 (35.4%)	
Moderate, satisfactory	412 (62.6%)	416 (61.6%)	
Poor, very poor	27 (4.1%)	20 (3.0%)	

^*^ χ^2^-test.

## Discussion

Women participating in the EPHT Trial were generally younger than those in the WHI and WISDOM trials [2,7,8,10]. The results from these trials show that HT improves specific symptoms of menopause. However, no effect of HT on self-rated health status or any other psychological (anxiety, depressed mood, memory/concentration) or somatic symptom was observed.

The mean age at recruitment in the EPHT trial was 59 years, whereas in the WISDOM trial it was 64 years [7]. The response rate in the EPHT trial was 75% compared to the 57% in the UK trial. The same WHQ questionnaire was used both in the EPHT trial and in the WISDOM trial to assess health related QOL. Combined HT started after menopause reduced vasomotor symptoms, sexual and sleep problems both in the WISDOM trial and in the EPHT trial after one year of use. Similarly in both trials, no significant differences were found in other menopausal symptoms, depression, or overall quality of life [7].

The mean age in the WHI trial at recruitment was 63 years. The response rate to different questions ranged from 87% to 90% in the WHI trial. QOL was assessed with the use of the RAND 36-item health survey. In the WHI trial, more women assigned to combined HT and symptomatic at baseline reported relief of hot flashes, night sweats, vaginal dryness at the end of the first trial year, but women in HT arms were more likely to develop breast tenderness, vaginal discharge, and headaches [2]. HT had no effect on depression, somatic symptoms, memory, or anxiety [2]. The use of HT was not associated with a meaningful benefit in QOL; there was only a small benefit in terms of sleep disturbance, physical functioning, and bodily pain after one year in trial [8]. Among women 50 to 54 years of age with moderate to severe symptoms at baseline, combined HT improved vasomotor symptoms and had a small benefit in terms of sleep disturbance, but not in other quality-of-life outcomes [8].

The mean age of women participating in the PEPI Trial was 56.1 years at baseline, and 52 symptoms were self-reported according to a check-list at year one and three. The 875 women participating in the trial were assigned to one of the five trial arms, and analysis was restricted to adherent women only. No baseline assessment of symptoms was reported. The results from the PEPI trial suggested a protective effect of postmenopausal HT for vasomotor symptoms, an increase in breast discomfort among users of progestin-containing regimens, and little influence on anxiety, cognition, or affect [9].

Thus the results of the current study add to the findings of previous research and confirm the pattern of results that appears to be consistent for a range of ages of women starting HT. The current EPHT results are also similar to those reported after the second trial year of the EPHT trial except for painful intercourse [12]. At the end of the first trial year, painful intercourse was improved by HT, whereas at the end of the second trial year, the effect was reversed [12]. A possible reason for this could be the decrease in adherence rates between the first and second years; at the end the first trial year, the proportion of women taking more than 80% of allocated drugs in treatment arms was 46% whereas at the end of the second trial year it was 32% [11]. All analyses were done according to intention-to-treat principle.

The WHQ was used in the Estonian language for the first time, and it proved to be a useful and sensitive tool to assess quality of life in this specific age group of women. In contrast the EuroQoL questionnaire, which measures generic health related QOL, that was used at the end of the second trial year of the EPHT trial did not detect any differences between the trial arms [12]. Including both menopause specific and generic QOL measures is recommended for quality of life measurements in this target group.

To examine the impact of blinding on recruitment, randomisation occurred before signing the informed consent. A large number of randomised women declined their participation and did not enter the trial, but since this happened before recruitment, it could have not been related to their treatment allocation. At recruitment, there were no baseline differences in the prevalence of symptoms during past two weeks in different trial arms except for sweating which was reported more often by women in the non-blind HT arm than in the non-treatment arm [12]. There were no differences between background characteristics of the women in four trial arms [10,12]. One of the limitations of this study is that QOL was not assessed at baseline. We presume that there were no significant differences between the trial arms in that aspect due to randomisation. Low adherence rates may have diluted to some extent the differences between the trial arms.

The differences in the results of the menstrual problems (WHQ subscale) between HT and non-HT groups at one year are likely to reflect vaginal bleeding, caused by HT. Therefore, they were not regarded irrelevant for postmenopausal women and were included in the present analysis. Previous analysis showed that HT increased the risk of bleeding substantially [12]. Our questionnaire did not include questions on other adverse events of HT, such as breast tenderness, but these have been the most often cited reasons for non-adherence to HT in earlier studies [11].

## Conclusions

In the light of previous research in this area, the results from the EPHT trial confirm that HT is not justified for treating symptoms other than vasomotor symptoms among postmenopausal women. Our study shows that many symptoms that are often attributed to menopause, such as psychological and physical symptoms, are not changed by HT and therefore HT should not be recommended as a treatment for these symptoms which are likely to have other causes.

WHQ allowed a detailed insight on various aspects of life quality of in this target group. It could be used more widely in evaluations of medical and non-medical interventions among peri- and postmenopausal women.

### Availability of supporting data

Advantages and disadvantages of postmenopausal hormone therapy: a preventive trial - the Estonian Postmenopausal Hormone Therapy trial. http://www.controlled-trials.com/ISRCTN35338757/35338757

Hovi Sirpa-Liisa. Preventive Trial on Postmenopausal Hormone Therapy in Estonia. A study of treatment preferences and trial process within a changing environment. http://www.stakes.fi/verkkojulkaisut/muut/Tu157Stakes2006.pdf

Veerus Piret. The impact of postmenopausal hormone therapy on health and use of health services: experience from the Estonian Postmenopausal Hormone Therapy (EPHT) trial. http://acta.uta.fi/pdf/978-951-44-7134-6.pdf

## Authors’ contributions

PV accepts full responsibility for the work and for the conduct of the study, had full access to all data, and controlled the decision to publish. PV, SLH and EH are responsible for the study concept and design, PV and SLH for acquisition of data. TS, MH, PV, SLH and EH contributed to the data analysis and interpretation of data, SLH and PV drafted the manuscript and all authors read and approved the final manuscript.

## Competing interests

All authors declare that they have no competing interests.

## Details of Ethics Approval

The trial design was approved by Tallinn Medical Research Ethics Committee, Estonia on January 22, 1998 (approval No 2). All participants gave written informed consent.
